# The Recovery concept in Assertive Community Treatment: Truth or Fake?

**DOI:** 10.1192/j.eurpsy.2024.1488

**Published:** 2024-08-27

**Authors:** J. J. Martínez Jambrina, L. I. Fernández, A. R. F. García, C. C. González, C. P. Martínez, A. F. Llorca, R. A. Díaz, F. V. Ortola, N. S. Guzmán

**Affiliations:** Psychiatry, Hospital San Agustín, Avilés, Spain

## Abstract

**Introduction:**

The concept of “Recovery” in the context of psychiatric rehabilitation has undergone significant evolution throughout history. This abstract delves into the question of the truth or falsity of this concept, examining diverse perspectives and arguments surrounding its application.

**Objectives:**

The primary aim of this abstract is to critically analyze the concept of “Recovery” in psychiatric rehabilitation and ACT from both favorable and critical perspectives, considering its historical evolution, and highlighting key distinctions between the theories of Mike Slade and William Anthony.

Furthermore, it addresses the significance of measuring and evaluating the fidelity of healthcare practices to this mode

**Methods:**

To conduct this analysis, an exhaustive review of current scientific literature was undertaken.  Emphasis was placed on the importance of measuring and evaluating the fidelity of healthcare practices to this model.

**Results:**

Slade and Anthony’s theories emphasize different aspects of recovery, while implementation models translate these theories into clinical practice and services. Additionally, the discussion highlights the significance of measuring and evaluating the fidelity of healthcare practices to this model.

Assertive Community Treatment (ACT) programs have increasingly recognized the importance of the “recovery” concept in promoting the empowerment and self-determination of individuals with severe mental illnesses. This discussion examines how ACT programs have adopted recovery-oriented principles, the ways in which they implement these principles, and the potential benefits and challenges associated with their integration.

**Conclusions:**

The distinctions between Mike Slade and William Anthony’s theories and the implementation models underscore the importance of a precise and differentiated understanding within the field of psychiatric rehabilitation.

The integration of the “recovery” concept within Assertive Community Treatment (ACT) represents a significant shift towards person-centered care in psychiatric rehabilitation. Further research and evaluation are essential to assess the effectiveness and long-term impact of this integration.

**References:**

1. Anthony, W. A. (1993). Recovery from mental illness: The guiding vision of the mental health service system in the 1990s. Psychosocial Rehabilitation Journal, 16(4), 11-23.
2. Slade, M. (2009). Personal recovery and mental illness: A guide for mental health professionals. Cambridge University Press
3. Kortrijk, H. E., Mulder, C. L., Drukker, M., Wiersma, D., & Duivenvoorden, H. J. (2020). The effects of assertive community treatment on service use in a homeless population in the Netherlands: A randomized controlled trial. Administration and Policy in Mental Health and Mental Health Services Research, 47(3), 378-387

**Disclosure of Interest:**

None Declared

